# Alternate Hosts of *Puccinia striiformis* f. sp. *tritici* and Their Role

**DOI:** 10.3390/pathogens9060434

**Published:** 2020-06-02

**Authors:** Sajid Mehmood, Marina Sajid, Jie Zhao, Lili Huang, Zhensheng Kang

**Affiliations:** 1 State Key Laboratory of Crop Stress Biology for Arid Areas, College of Plant Protection, Northwest A&F University, Yangling 712100, China; sajid.mehmood@nwsuaf.edu.cn (S.M.); jiezhao@nwsuaf.edu.cn (J.Z.); 2 College of Food Science and Engineering, Northwest A&F University, Yangling 712100, China; msajid1118@nwsuaf.edu.cn

**Keywords:** *Puccinia striiformis* f. sp. *tritici*, alternate hosts, stripe rust of wheat, genetic diversity, epidemics

## Abstract

Understanding the interactions between the host and the pathogen is important in developing resistant cultivars and strategies for controlling the disease. Since the discovery of *Berberis* and *Mahonia* spp. as alternate hosts of the wheat stripe rust pathogen, *Puccinia striiformis* Westend. f. sp. *tritici* Erikss. (*Pst*), their possible role in generating new races of *Pst* through sexual reproduction has become a hot topic. To date, all the investigations about the role of alternate hosts in the occurrence of the wheat stripe rust epidemics revealed that it depends on alternate host species and environmental conditions. In this review, we summarized the current status of alternate hosts of *Pst*, their interactions with the pathogen, their importance in genetic diversity and disease epidemics. Most importantly, the recent research progress in understanding the role of alternate hosts of *Pst* is provided.

## 1. Introduction

Wheat is the most cultivated cereal crop, universal staple food, and a host for many pathogens. The most serious threat to wheat crops is a group of rust fungi causing severe yield losses worldwide [1]. Stem rust or black rust, caused by *Puccinia graminis* f. sp. *tritici* Erikss. and E. Henn. (*Pgt*), leaf rust or brown rust, caused by *Puccinia triticina* Erikss. (*Pt*) and the wheat stripe rust or yellow rust, caused by *Puccinia striiformis* Westend. f. sp. *tritici* Erikss. (*Pst*), is a historically crucial economic disease that occurs in almost all wheat-growing regions worldwide [2,3]. The early history of mankind is full of fears and threats to these devastating rust pathogens. Since the discovery of rust pathogens, numerous investigations have been conducted on their life cycles for the management of the diseases caused by these fungi. The tenacity of rust fungi as destructive pathogens throughout the wheat-growing areas in the world is attributed to the special features of the pathogen, for example, the production of a large number of spores, inter and intracontinental wind dissemination, and the ability to change genetically resulting in new races with increased virulence diversity [4]. Generally, the disease occurs in the northern and southern areas of temperate regions. Recently, the wheat stripe rust disease has become more severe in some warmer areas than before [5], endangering global food security [6]. Hot summers and dry seasons are the bottlenecks for the survival of *Pst*. The disease can be controlled by growing resistant cultivars, suitable cultural practices, and the appropriate use of chemical fungicides. Resistant cultivars are the most effective, economical and environmentally friendly approach to combat with the wheat stripe rust pathogen. However, the *Pst* population is highly dynamic and variable, which makes it difficult to develop highly resistant wheat cultivars with durable resistance [6]. 

In the US Pacific Northwest, barberry is essential for the wheat stem rust but does not play a role for the wheat stripe rust pathogen [7,8,9]. Barberry may serve as an alternate host for *Pst* in the Himalayan region under natural conditions [10,11,12]. In eastern Africa and western Asia, barberry plants have been found, but their association with the wheat stripe rust disease epidemics has not been confirmed [13,14]. To date, the evidence of natural infection of barberry by *Pst* has been observed only in China, but at a low frequency [15,16]. Similarly, *Pst* has not been found on barberry plants in southeastern Sweden, but *Pgt* is common on the alternate host plants in this region [17].

The use of genetic techniques in the past ten years has achieved some advancement in understanding the plant–microbe interaction. The wheat stripe rust pathogen is an obligate, biotrophic parasite, having five distinct spore stages and two hosts to complete their life cycle. A macrocyclic life cycle comprises of uredinial, telial, basidial, pycnial and aecial stages. Like other rust pathogens, *Pst* is also highly specific to their primary host plants, for example, cereal crops and grasses, and the alternate host plants, for example, *Berberis* and *Mahonia* spp. The primary hosts can be the same but generally, the alternate hosts are different for different *Puccinia* spp. Based on their host specificity and morphological characteristics, the *Puccinia* spp. are further divided into formae speciales or varieties. For example, stripe rust on wheat is caused by *P*. *striiformis* f. sp. *tritici* (*Pst*), on barley by *P*. *striiformis* f. sp. *hordei* (*Psh*); stem rust on wheat by *P*. *graminis* f. sp. *tritici* (*Pgt*), and on oat by *P*. *graminis* f. sp. *avenae* (*Pga*), and on rye by *P*. *graminis* f. sp. *secalis* (*Pgs*); and crown rust on oat by *P*. *coronata* var. *avenae* (*Pca*), and on barley by *P*. *coronata* var. *hordei* (*Pch*) [18].

The genetic diversity of *Pst* in Australia, Europe and North America indicated a clonal population structure of the pathogen [19]. On the other hand, the *Pst* populations of Gansu Province, China, were found to have high genetic diversities and produce abundant telia, indicating possible sexual recombination in this region [20,21]. Jin et al. [13] reported barberry as an alternate host for *P. pseudostriiformis* (Syn. *P. striiformis* f. sp. *poae*) under natural conditions in Minnesota in the US and *Pst* under controlled conditions. The possible role of *Berberis* spp. as a sexual host of *Pst* has attained much importance, particularly in the US, China, and Pakistan [7,9,11,15,18]. *Mahonia aquifolium*, under experimental conditions, has also been identified to be susceptible to *Pst* [22].

Based on the different effects of the microorganisms on plants, their relationship can be pathogenic, saprophytic or beneficial. However, in all types of interactions with plants the pathogens use similar strategies and mechanisms of the gene-for-gene model: for each gene of resistance in the host plant, there is a corresponding gene of virulence in the pathogen, and for each gene of virulence in the pathogen, there is a corresponding susceptible gene in the host plant. The wheat stripe rust-pathosystem follows the gene-for-gene concept [23]. An incompatible interaction (or resistance) is observed when rust isolates having an avirulence gene come across a host plant having a corresponding resistance gene. Based on this concept, it is presumed that avirulence is dominant over virulence for *Pst* [24]. A compatible or susceptible reaction is detected when a resistance gene does not function or is absent in the host plant. The nature of the host plant resistance and the capability of the pathogen to cause infection are important factors in pathogenesis. The most economical and preferred strategy to control *Pst* is the use of genetically resistant *R* genes. Seedling resistance genes confer resistance encoding nucleotide-binding site-leucine-rich repeat (NBS-LRR) R proteins that identify effector proteins, present in the cytoplasm and they stop pathogen multiplication by triggering a defense response [25,26]. Computational estimation is that *Pst* genomes encode over 1000 candidate effectors [27,28]. For the wheat chromosomes, hundreds of disease resistance (*R*)-gene loci have been genetically mapped, but only a small number of stripe rust resistance genes (*Yr*) have been isolated to date, for example, *Yr7*, *Yr10*, *Yr15*, *Yr18*, *Yr36*, *Yr46*, and *Yr5*/*YrSP*, [29,30]. *Yr*10 is the best example of a cloned *R* gene against *Pst,* which confers resistance to many *Pst* isolates worldwide. However, several *Pst* isolates virulent to *Yr*10 have been identified [31]. The successful cloning of *Yr15*, another broad-spectrum *R*-gene discovered in the 1980s, derived from wild emmer wheat, encoding a putative kinase-pseudokinase protein, nominated as wheat tandem kinase 1, comprising a unique *R*-gene structure in the wheat, was reported by Klymiuk et al. [29]. Although some *Pst* races have become virulent to *Yr15* in the 2000s [32], with time, the evolution of new virulent isolates of *Pst* is a characteristic of race-specific resistance genes, known as the boom and bust cycle. Adult plant resistance genes delay pathogenic infection and spore production. These non-classical *R* genes cause resistance allele-specific protein variants, molecularly unrelated to NBS-LRR proteins [25]. *Yr18*, *Yr46*, and *Yr36* are good examples of these resistance genes [25,33,34]. The strategy to combat *Pst* using a combination of different types of resistance genes has been found to be very useful because it slows down the life cycle of the pathogen and reduces its population size. However, the durability of these genes may be affected by the worldwide genetic diversity of the pathogen as a result of asexual or sexual recombination [35]. 

The breeders and pathologists strongly believe in the genetic control of this disease using yellow rust resistance genes (*Yr*), and they have been working on the identification of such resistant genes over the last 100 years [36]. In recent years, the use of DNA-based technology has made it possible to clone first *Yr* resistance gene, the identification of the complete life cycle of the wheat stripe rust pathogen, the identification of the center of diversity in the Himalayan region, their past global migration routes and patterns, and most importantly the provision of the drafting of *Pst* genomes [35]. In this review, we focused on (i) the research progress on the identification of alternate hosts to *Pst* in the world; and (ii) their possible role in the pathogenic diversity; (iii) recent research progress in understanding the genetics of *Pst*.

## 2. Stripe Rust: Outlook

Wheat rusts have created major famines throughout history, causing substantial economic losses [37]. Currently, the most severe rust disease is the wheat stripe rust disease, causing more than 60% yield losses under favorable conditions [1,25,38]. The disease is named as yellow rust or stripe rust due to yellow colored stripes in lines between leaf veins in adult-plants but the urediniospores are in clusters (not in stripes) when the infection is at the seedlings stage [39]. Urediniospores are dikaryotic and produced asexually on the primary host plant. In the case of severe disease epidemics, stripe rust uredinial infection occurs on leaves, spikes, spikelets, glumes, awns and kernels. With the increase in temperature or at the maturity stage of the plant, the production of urediniospores comes to an end. The uredinia start converting into black colored telia containing teliospores [40]. Teliospores may serve as the survival structures which can cause infection on the alternate hosts under favorable environmental conditions or become a dead-end due to severe climatic conditions or incompatibility with the alternate host plants [41]. Teliospores are thick walled and germinate to produce haploid basidiospores [18]. These basidiospores directly penetrate the alternate host epidermal cells and cause infection and produce pycnia on the upper side of leaves and aecia on the lower side of leaves. Under favorable conditions both the pycnial and aecial infections are observed on stems, pedicels and peduncles [12]. The aeciospores infect wheat crops resulting in the formation of urediniospores. In contrast to uredinial re-infection on the same primary host plants or grasses, the aeciospores cannot re-infect the alternate host plants. 

The wheat stripe rust pathogen belongs to the genus—*Puccinia*, family—Pucciniaceae, order—Pucciniales, class— Pucciniomycetes, division—Basidiomycota and kingdom—Fungi. *Pst* can undergo long-distance dispersal and it has caused numerous invasions [42,43] associated with austere economical losses [19,44,45,46]. Several cases of incursions of economic importance have been reported for *Pst* but only recently their origin was confirmed [11,43]. In the early 20th century, *Pst* was reported for the first time in South and North America [47,48], most likely spreading from north-western Europe [11,45] and introduced accidentally in Australia from north-western Europe in 1979 through human activity [49]. *Pst* strains detected in South Africa in 1996 were genetically related to populations in the Mediterranean regions and Middle Eastern ones, possibly spread by the wind [11,50]. *Pst* has become important in the context of invasions and recolonizations through the emergence of new races and strains in previously non-colonized areas. For example, since 2000, the emergence of two aggressive strains of *Pst*, PSTS1, and PSTS2, in the geographical expansion of *Pst* epidemics into the southeastern US and western Australia, where the disease was not previously a serious problem [40,51]. Similarly, since 2011, invasive strains of the wheat stripe rust pathogen, Warrior and Kranich, have largely replaced the pre-existing northwestern European *Pst* populations of the pathogen [43,52].

*Pst* has been reported in the areas of the US (Pacific Northwest), eastern Asia (northwestern and southwestern China), Oceania (Australia, New Zealand), southern Asia (India, Pakistan, and Nepal), western Europe (eastern England), the Arabian Peninsula (Yemen) and eastern Africa (Ethiopia, Kenya) [53]. In the last two decades, the emergence of more aggressive races of *Pst* having the ability to cause high epidemic potential even in warmer regions [39] is generally the result of mutation, somatic hybridization and sexual recombination. The potential role of alternate hosts in pathogenic diversity is of much importance. However, it is still unknown by which mechanisms new races evolve. The high reproduction ability, long distance dissemination, adaptation to different environmental conditions, and several host species make *Pst* a highly diversified pathogen [54]. The threat of new virulent races of this pathogen emphasizes the need to understand the mechanisms involved in the genetic diversity of *Pst* and the role of aecial hosts in sexual reproduction to encounter the possible attacks of *Pst* in the future [39]. 

## 3. The Life Cycle of *Pst*

The complete life cycle of *Pst* has five spore stages, different from each other, on two phylogenetically distinct host plants, a cereal as the primary host or asexual host, and *Berberis* spp. as the alternate host or sexual host [13]. The dikaryotic (N + N’) single-celled urediniospores appear on the primary host through the breaking of epidermal cells, and each uredinia harboring yellow-colored numerous urediniospores. The repeated asexual cycles on the primary host may cause wide-scale epidemics on the cereal hosts [55]. The single uredinia, assembled in lesions, forms typical stripes on the leaves of adult plants and produce urediniospores in 10 to 18 days after infection under optimal conditions. The uredinial lesions expand longitudinally upon the production of new uredinia. With the start of the senescence of infected leaves, *P. striiformis* starts producing telia resulting in the creation of many two-celled oblong-clavate teliospores. These teliospore cells contain a diploid nucleus (N + N’) formed by karyogamy. The germinating teliospores produce ellipsoid haploid (N) basidiospores. Basidiospores are uninucleate or binucleate haploid spores produced from a germinating diploid teliospores. Basidiospores cause infection on the alternate host (e.g., barberry), resulting in oblong-shaped pycniospores (N) on the adaxial surface of the leaf, following the formation of dikaryotic (N + N’) aecia on the abaxial surface of the leaf. Pycniospores are haploid (N), sexually derived spores (spermatium) formed in a pycnium (spermogonium) of rust fungi. Finally, aeciospores infect the primary host resulting urediniospores into the wheat leaves. The life cycle of *Pst* may take place during two regular growing seasons of the asexual host. *Pst* is an obligate biotrophic fungus which depends on a living host for its development and reproduction [24,45,55].

**Figure 1:**
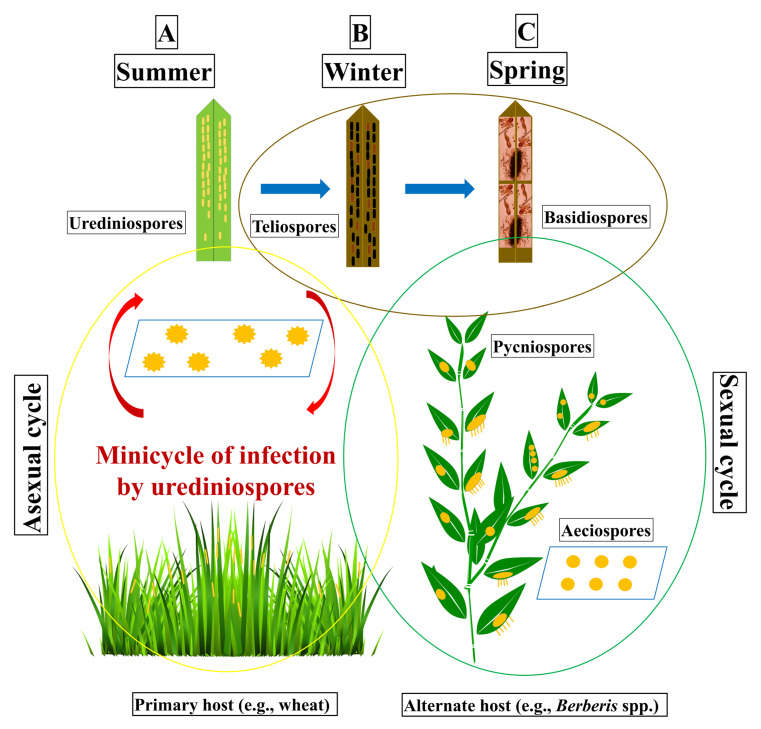
Schematic diagram of the life cycle of *Puccinia striiformis* f. sp. *tritici* (*Pst*), divided into three phases from left to right. Phase **A**, usually starts late in the spring and continues into the summer until the harvesting of the wheat crop. It consists of the asexual cycle which takes place on the primary host (e.g., wheat). The infection may take place by aeciospores or urediniospores resulting in the formation of yellow-colored stripes of uredinia on the wheat leaves. Urediniospores re-infect wheat plants in the same field or in the neighboring wheat fields to continue the mini-cycle of somatic reproduction until the conditions become unfavorable. With the increase in temperature or at crop maturity, the urediniospores turn into teliospores. Teliospores are thick-walled resting spores. Phase **B**, the teliospores survive and germinate to produce basidiospores. Phase **C**, the basidiospores infect the new leaves of alternate hosts (e.g., barberry) in the spring and produce pycniospores on the adaxial surface of leaves. Pycniospores produce aecial cups on the abaxial surface of the leaves after fertilization. Aecial cups contain aeciospores which can infect the primary hosts.

The sexual phase starts when the two-celled teliospores germinate and produce basidiospores, attached to a sterigmatum. Basidiospores (N) infect barberry leaves, resulting in the formation of pycnia (N), covered with pycnial nectar, formed on the upper side of the leaf. The haploid pycniospores of (−) and haploid hyphae (+) fuse together through plasmogamy and form aeciospores (N + N) on the abaxial side of the barberry leaf [56]. Aeciospores are dikaryotic produced in a cup-shaped aecium of a rust fungus. The asexual infection process in cereal hosts starts via a urediniospore-germ-tube, penetrating a stoma and then differentiating into a substomatal vesicle, resulting in two to three primary infection hyphae, which develop haustorial mother cells. These cells are separated from their respective hyphae by a septum. Haustorial mother cells penetrate the plant cell walls and form haustoria, highly specialized structures, representing the primary interface between host and the pathogen. Haustoria take water and nutrients from the host tissues and also make signaling between hosts and pathogens by producing effector molecules, like avirulence gene products. Young haustoria have a spherical shape, whereas older haustoria appeared more branched which allows the fungus to extend the area of the contact zone into the host and uptake nutrients more efficiently [45]. Basidiospores can only infect alternate hosts (like barberry) but cannot infect primary hosts (like wheat). Basidiospores infect epidermal cells through direct penetration while urediniospores infect through host stromata. *Pst* generally infects common wheat (*Triticum aestivum* L.), cultivated emmer wheat (*T. dicoccum* Schrank), triticale (*Triticosecale*), durum wheat (*T. turgidum* var. *durum* L.) and wild emmer wheat (*T. dicoccoides* Korn); as well as cultivated barley (*Hordeum vulgare* L.) and rye (*Secale cereale* L.). The complete life cycle of *Pst* is shown in Figure 1. 

## 4. Current Research Status of the Alternate Hosts of *Pst*

**Figure 2:**
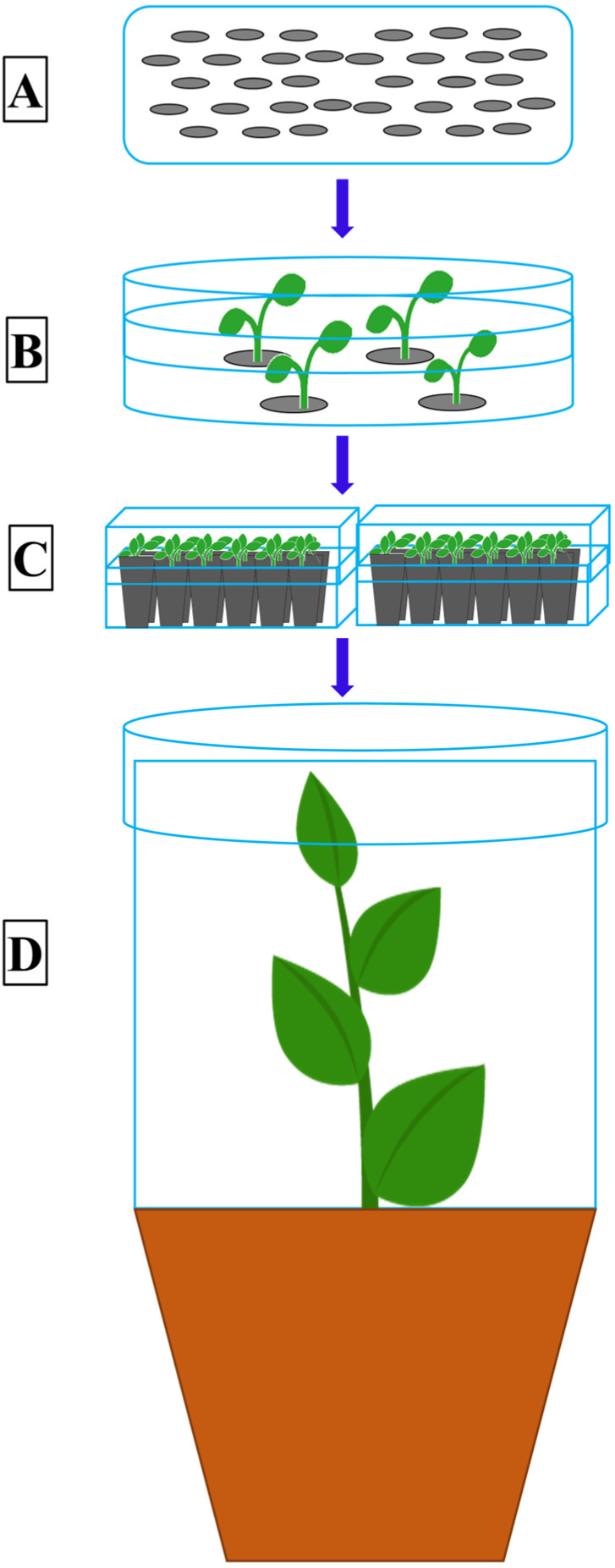
The general procedure to grow barberry seedlings from seeds under greenhouse conditions. **A**, the mature and dried seeds collected from naturally growing barberry plants. **B**, 15–20 seeds placed on the blotter paper moistened with distilled water in a plastic Petri-plate. These Petri-plates are incubated in a growth incubator at 10 ℃ temperature, 12/12 hrs light/dark period, and 100% relative humidity (RH). Seeds are moistened every 2–3 days until the seedlings start emerging from the seeds. **C**, at two to the three-leaf stage, the seedlings are gently transferred to plastic potted plant trays having been filled with a mixture of half medium-grit sand and half peat moss. The trays are covered with a plastic lid and again placed into the growth incubator at the same conditions of light, temperature and moisture. **D**, at the 3–4 leaf stage, the barberry seedlings are transplanted into bigger plastic pots (10 × 9 × 7 cm) filled with potting soil mixture (2 parts compost; 2 parts peat moss), with one plant per pot, and kept in a growth chamber at 20–22 ℃, 16/8 h light/dark cycle, and 60–75% relative humidity (RH). The young plants are watered as needed for good growth.

**Figure 3:**
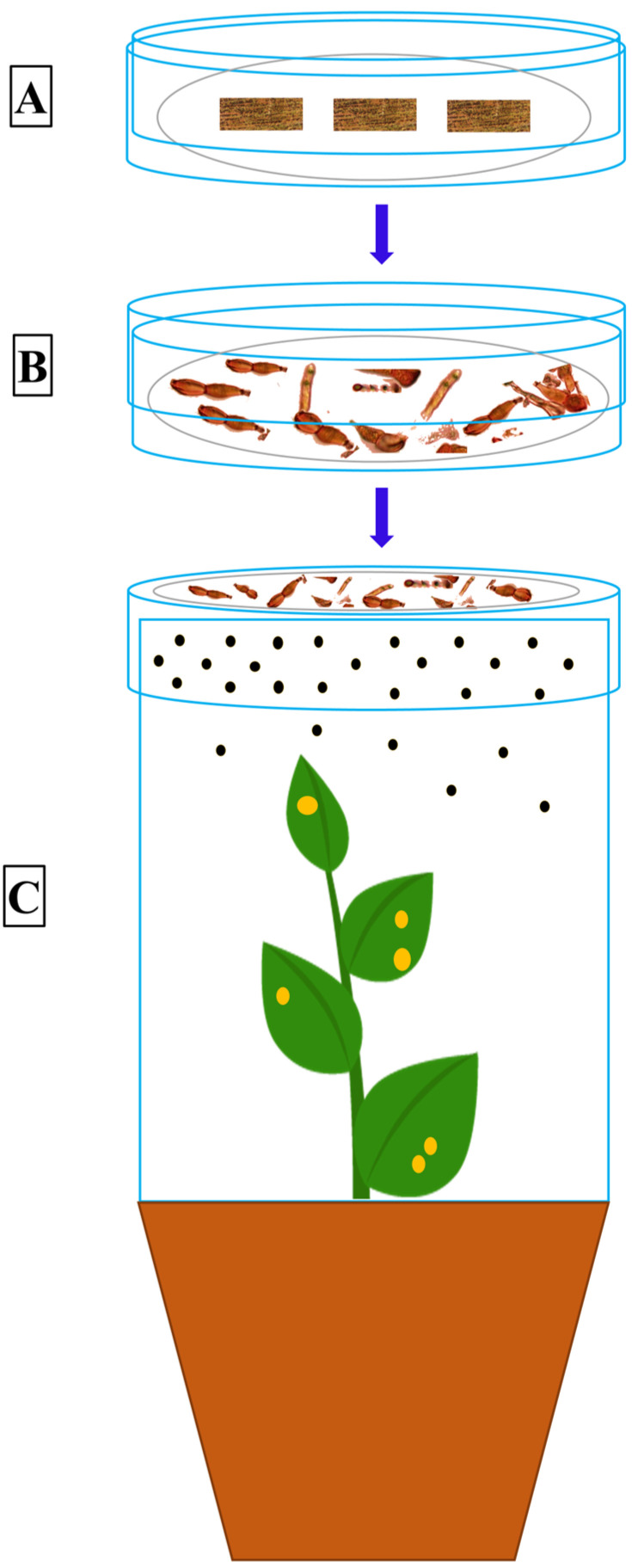
The general technique of artificial inoculation, using wheat leaf segments containing mature telia, is used to check the susceptibility of barberry species against *Puccinia striiformis* f. sp. *tritici* (*Pst*), and to generate selfing or crossed progeny populations using a single isolate or isolates of the *Pst* pathogen under controlled conditions. **A**, the wheat leaf segments bearing *Pst* telia are soaked in distilled water in a Petri-dish (25 cm in diameter) and kept at room temperature (25–26 °C) for two days. After rinsing with distilled water, the leaf segments are placed on 2% water agar media and incubated at 10 °C in dark. **B**, the teliospores germination is examined using a light microscope. **C**, when abundant basidiospores are observed (usually from 1 to 2 days after planting), the water agar plates containing basidiospores are inverted and placed on the top of a plastic cylinder surrounding the barberry plants bearing young leaves (from 10 to 15-days-old). The inoculated barberry plants are incubated at 100% RH for three days at 10 °C in the dark and then kept in a spore-proof growth chamber with 90% to 100% RH, the diurnal cycle of 16/13 °C, and 12/12 h light/dark cycle to promote the pycnial formation. Plants are observed for symptoms and signs and misted with water every day until the pycnia appear (from 12 to 14 dai) on the adaxial surface of the leaves. Pycnial nectar is picked from one pycnium and delivered to another using a sterilized toothpick for fertilization. At about 18–22 dai, when aecia appear on the abaxial surface of leaves, the RH is lowered to 60% to 70% to stop the opening of the aecial cups.

**Figure 4:**
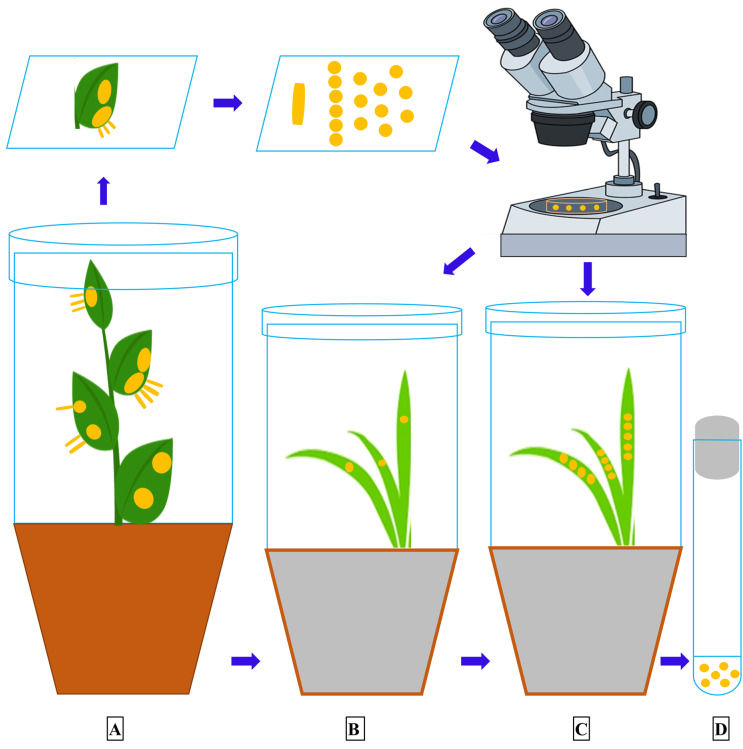
The procedure to inoculate wheat seedlings with aeciospores under controlled greenhouse conditions. **A**, an artificially inoculated barberry plant having aecial cups on the abaxial surface of the leaves. Aecial cups (1–3 mm in length) are used to produce inoculum, cut with a sterile blade and placed in a drop of deionized water on a glass slide and crushed gently with a needle to release the aeciospores. A sterile needle is used to inoculate 10-day wheat seedlings with aeciospore suspension. **B**, the inoculated seedlings are incubated for 24 h in a dew chamber at 10 °C and then transferred to a growth room at 16 °C with a diurnal cycle of 16/8 h light/dark. The inoculated seedlings are isolated with plastic cylinders with open tops to prevent contamination. The plants are checked for symptoms and signs at about 12 days and uredinial sporulation is recorded 15–20 days after the aeciospore inoculation. **C**, the susceptible wheat seedlings inoculated with aeciospores from the tested *Berberis* spp. produce typical *Pst* urediniospores 15–20 days after inoculation. **D**, the urediniospores are collected in a lab test tube. For further experimentation, the urediniospores are stored at 4 °C for a short period (less than 2 months) and at −80 °C for a long time [12].

An alternate host is a plant, entirely different from the primary host plant, on which a rust fungus can develop and complete its life cycle. All the early efforts to find alternate hosts for *Pst* were unsuccessful [44,57,58,59], however Mains [60] made the correct speculation, proved after 70 years, about the possibility of *Berberis* and *Mahonia* spp. as the alternate hosts of *Pst* due to their similarity with *P. arrhenatheri*, *P. koeleriae* and *P. montanensis*. The complete life cycle of the wheat stripe rust pathogen was demonstrated by Jin et al. [13] declaring some *Berberis* spp. as the alternate hosts for the wheat stripe rust pathogen. After this discovery, Wang and Chen [22] inoculated *Mahonia aquifolium* (Oregon grape) under controlled greenhouse conditions and showed *M*. *aquifolium* as an aecial host for *Pst*. Similarly, Zhao et al. [15,18] identified several barberry species as the alternate hosts of *Pst* in China through artificial inoculation. Mehmood et al. [12] inoculated seven barberry species collected from the Himalayan region of Pakistan and found them susceptible hosts to *Pst* under controlled conditions. Similarly, Zhuang et al. [61] and Du et al. [62] identified three and ten *Berberis* species, respectively, from China under controlled conditions. The general procedure adopted for growing barberry seedlings and the artificial inoculation technique, using germinating teliospores, for the identification of sexual hosts of *Pst* under controlled conditions is described in the schematic Figure 2 and Figure 3. The schematic diagram showing the procedure to inoculate the wheat seedlings with aeciospores from artificially inoculated barberry is described in Figure 4.

The detection of the alternative hosts of *Pst* was essential for a better understanding of the pathogen lifecycle and the relationship between the virulence of the pathogen and the relative degree of resistance of the host plant. *Berberis* spp. have been well known alternate hosts for *Pgt* for more than a century [63]. Before the discovery of *Berberis* spp. as a sexual host of the wheat stripe rust pathogen, it was challenging to study the virulence variation of *Pst*. Grasses and cereals rust fungi have ≥380 species in the genera of *Uromyces* and *Puccinia*, are *heteroecious* [64]. *Puccinai striformis* is a complex species and it was reported to have five formae speciales based on host specialization, namely *Pst* on wheat, *P*. *striiformis* f. sp. *hordei* (*Psh*) on barley, *P*. *striiformis* f. sp. *agropyri* (*Psa*) on Agropyron spp., *P*. *striiformis* f. sp. *elymi* (*Pse*) on *Elymus* spp., and *P*. *striiformis* f. sp. *secalis* (on rye) [57]. Using the combined techniques of spore morphology and sequences of the internal transcribed spacer (ITS) and beta-tubulin DNA regions, the stripe rust pathogen infecting bluegrass and orchard grass were renamed as *P*. *pseudostriiformis,* M. Abbasi, Hedjaroude, and M. Scholle, and *P*. *striiformoides* M. Abbasi, Hedjaroude, and M. Scholle, respectively [65,66]. *P*. *striiformis* on grass species in the genera of *Hordeum*, *Aegilops*, *Elymus*, and *Triticum*, must be documented as *P*. *striiformis* sensu stricto [65] up to the finding of sexual hosts for *Pst* and *P*. *pseudostriiformis* (syn. *P*. *striiformis* f. sp. *poae*) on *Poa pratensis* [13]. 

The *Berberis* and *Mahonia* species are extensively distributed around the world except Australia. A total of 600 species (500 species of *Berberis* and 100 species of *Mahonia*) have been documented to date [12,54]. Half of the total numbers of *Berberis* and *Mahonia* spp. are said to be native to China [54]. The highest numbers of *Berberis* spp. are recorded in Asia as compared to the other six continents. *B. vulgaris* L. (also known as European barberry or common barberry), belonging to the kingdom Plantae—plants, subkingdom—Tracheobionta-Vascular plants, super-division—Spermatophyta-seed plants, division—Magnoliophyta-flowering plants, class—Magnoliopsida-dicotyledons, subclass—Magnoliidae, order—Ranunculales, family—Berberidaceae-barberry family, genus—*Berberis* L. barberry, species—*Berberis vulgaris* L. 

**Figure 5:**
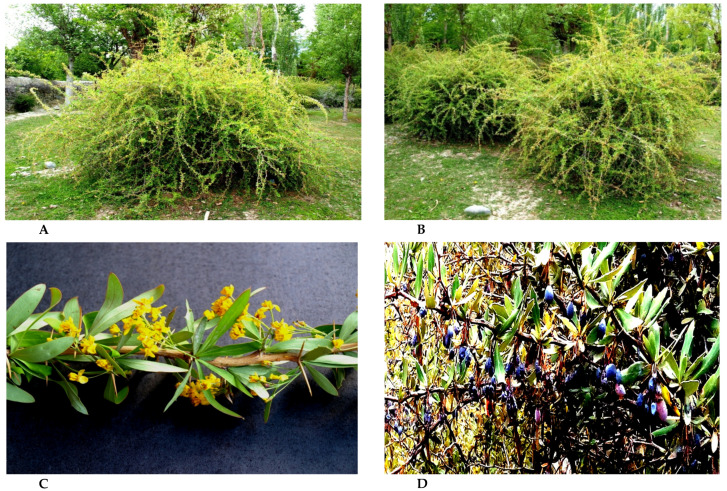
Barberry shrubs (*Berberis pseudumbellata*), **A** and **B**: growing naturally in the Himalayan region of Pakistan; **C**: inflorescence; and **D**: shoots with ripened berries [12].

Alternate hosts of the wheat stripe rust pathogen are considered to play an essential role in over-summering for the pathogen in adverse environmental conditions and offer new inoculum (generated through sexual recombination) for the development of wheat stripe rust disease epidemics. According to Zhao et al. [18], the function of an alternate host of cereal rusts depends upon the fungal species and ecological conditions for the disease epidemic and pathogenic diversity. Generally, an alternate host provides survival to the pathogen during adverse conditions serving as a sexual host and may generate diversified pathogenic populations through sexual recombination [14,67]. A representative shrub of barberry (*Berberis pseudumbellata*), showing inflorescence and shoots with ripened berries, naturally growing in the Himalayan region of Pakistan, is shown in Figure 5. Up until now, a total of fifty-three *Berberis* and *Mahonia* species susceptible to *Pst*, under controlled and natural conditions, have been identified. The list of alternate hosts (*Berberis* and *Mahonia* spp.) of the wheat stripe rust pathogen are given in Table 1.

## 5. Impacts on Genetic Diversity

The alternate hosts of *Pst* are known, but their specific role in the underlying mechanism of virulence diversity, phenotypic and genotypic changes and sexual recombination is not yet fully explored. The discovery of barberry as an aecial host of the wheat stripe rust pathogen suggests an essential role in the genetic diversity of the pathogen through sexual recombination. However, the inherent part of barberry species in sexual recombination causing genetic diversity is still unclear. Earlier studies [63,68] investigated the role of alternate hosts in generating virulence diversity of the stem rust pathogen. Environmental conditions, including weather conditions and cropping systems, are highly crucial for the wheat stripe rust disease epidemics. The disease development by urediniospores on cereal crops, and by basidiospores on alternate hosts, require special temperature and humidity. The environmental conditions not only affect the survival, infection, growth and reproduction of the fungi throughout the asexual cycle, but also affect the different stages of the sexual cycle, for example, the survival of teliospores, their germination and basidiospore infection on the alternate hosts [40,69,70,71,72].

Ali et al. [73] studied the temporal maintenance of *Pst* populations from the normal wheat-growing seasons (winter) to off-season (summer) in the Himalayan region of Pakistan. They proposed a sink and source relationship between non-*Berberis* and *Berberis* zones for the significance of the sexual cycle of *Pst* in the center of origin. *Pst* basidiospores could potentially infect barberry spp. in the Himalayan region if teliospores on wheat plants can germinate in the spring or late in the fall and remain throughout the dry winter as reported in Gansu, China [15,16]. Whether barberry plants provide aeciospores to start the wheat stripe rust disease in the Himalayan region needs further investigations. Identification of aecia on naturally infected barberry plants infecting wheat plants and using molecular markers [7] is the most direct and powerful approach to answer the questions whether and to what extent alternate hosts are important for the wheat stripe rust pathogen in the Himalayan region of Pakistan and other regions in the world.

The high genetic diversity for both the virulence and molecular markers were discovered in *Pst* populations in the Chinese Gansu Province and in the Middle East [20,21,74] which emphasizes the hypothesis of periodic recombination in these regions, even though the exact mechanism was not determined. The isolates from China are more readily produced telia than the isolates obtained from Europe, suggesting that the tremendous diversity in Gansu is due to sexual reproduction [10]. The results of these observations are in contrast to several studies in Europe [19,75], Australia [76] and the Yunnan area of China [77], where the genetic diversity was generally low and consistent with a clonal *Pst* population structure. These results show that the role of sexual recombination in *Pst* is different among regions, and depends on the opportunity for sexual reproduction and somatic hybridization. However, the details and the impact of both processes under natural conditions remain to be investigated.

## 6. Factors: Taxonomy, Morphology, and Phenology of Alternate Hosts

Taxonomic entities of susceptible alternate hosts are essential because these serve not only as a carrier but also as a breeder of possible new and aggressive strains of the wheat stripe rust pathogen. It is important to identify barberry species. Different investigations may give plants with the same morphology and physiology different names to one *Berberis* spp. Several questions are also related to the taxonomy of the barberry plants. Protocols for the identification of different *Berberis* spp. (such as photos, drawings, previous literature and web-based data) can be helpful for the taxonomical identification of barberry species in an area. However, the taxonomic classification of different barberry species is still controversial in some areas in the world (e.g., the Himalayan region in Pakistan) and it needs further investigations, preferably DNA-based technology to classify the barberry species/subspecies [78].

The phenology of alternate hosts and ecological surroundings are important factors to determine if *Pst* can infect primary and alternate hosts in a specific area [12]. Wang and Chen [9] reported that teliospores are produced on winter wheat (from June to July) and on spring wheat (from July to August) in the US Pacific Northwest. Under controlled conditions, the mature teliospores freshly harvested from wheat leaves can germinate readily. The germination rate will decrease over time. The precipitation in the region occurs mainly during the winter from November to February. From mid-April to early May, new leaves of barberry emerge, but at that time, teliospores are not viable, resulting in the absence of aecial infection. While the teliospores of *Pgt* are produced from June to July, they remain dormant until the winter ends. They start germinating in April to May and infect young leaves of susceptible barberry plants to cause infection in May and June. As a result of infection, aeciospores are released to infect cereal crops in June and July. Therefore, barberry in this region plays a vital role in *Pgt* but not for *Pst* [7,9]. Jin et al. [13] hypothesized that in areas where wheat and *Pst*-susceptible *Berberis* spp. coexist, sexual recombination likely plays an active role in contributing to the diversity of *Pst*.

Mehmood et al. [12] described that barberry plants produce new leaves from March to mid-April in the Himalayan region of Pakistan, while *Pst* telia are produced usually in late May to late June in the winter wheat areas. In the low elevations and in the spring wheat areas of high altitudes from October to November [73]. According to Wang and Chen [9] in areas having a dry winter and a wet spring (like northwestern China), there is a possibility that *Pst* telia can survive the winter and infect barberry plants. The climatic conditions of the Himalayan region are of such types that make *Pst* able to infect barberry plants in this region [12].

## 7. The Important Role of the Alternate Hosts

A main function of alternate hosts is helping *Pst* survive during adverse environmental conditions. For example, wheat is largely grown as a winter crop in the valleys of the Himalayan region in Pakistan to fulfill the requirements for human food and animal feed. In the lower parts (1200–1900 m above sea level) of valleys, a double cropping system is used for growing two staple crops, wheat from November to May and maize from June to October. In the areas of high altitudes (2300–3000 m above sea level), maize is the main crop grown from May to October or June to November and summer-sown wheat is grown as a secondary or minor crop [11,79]. In this region, both primary and alternate hosts coexist, an ecological condition required for *Pst* to complete the macrocyclic lifecycle on these distinct plants. 

One more important function of the alternate hosts of the wheat stripe rust pathogen is the production of new races with combined virulence genes through sexual reproduction as described in case of *Pgt* [80] *P*. *graminis* f. sp. *avenae* (*Pga*) [81] *P*. *coronata* f. sp. *avenae* [82] indicating that alternate hosts increase the number of races [83,84]. Similarly, Wang et al. [7] identified 10 races of *Pgt* from 16 single-uredinium cultures from aecia on barberry bushes in northern Idaho in the US.

Another important role of the alternate hosts of the wheat stripe rust pathogen is population diversification through sexual reproduction on its alternate hosts e.g., barberry. For example, the number of new races was significantly larger in the areas with the alternate hosts than the barberry eradication regions in the case of *Pgt*. A comparison of virulence phenotypes of *Pgt* [63] near barberry plants in eastern Washington and northern Idaho (reproduced sexually) and of populations east of the Rocky Mountains (reproduced asexually) resulted in the identification of 100 races (23.5%) out of 426 isolates from the sexual populations, and 17 races (0.07%) out of 2377 isolates from asexual populations. Similarly, in the US Pacific Northwest, high genetic diversity in the *Pgt* population has been observed in recent years [9,85]. High genetic diversity has also been reported for a sexually produced *P*. *cerealis* population [86]. Although a large number of rather rare races collected from the alternate hosts, or cereal and grasses near alternate host plants, have been reported for *Pgt*, indicating the importance of alternate hosts in generating diverse races, a lot of investigations are needed in the case of the wheat stripe rust pathogen especially for the barberry plants infected under natural conditions.

## 8. Research Progress; Revealing the Genetic Diversity of *Pst*

Alternate hosts are used to study the genetics of the rust pathogens. For example, the study of inheritance and virulence diversity in a *Pst* population is generated through artificial inoculations on barberry plants using the germinating teliospores of a dominant race of *Pst*, under controlled conditions [12,15,24,87,88,89]. Their pathogenicity characteristics and traits like latency, lesion size, spore production rate, sporulation duration, spore color and telial formation have also been considered [67]. The discovery of alternate hosts of the wheat stripe rust pathogen led to further investigations of the genetics of *Pst*. The most common approach to access and quantify the genetic factor involved in pathogenic diversity is to measure the value of the concordance information obtained from the individuals of a population. Researchers have utilized common barberry species as a model system for the genetic studies of *Pst*. Recently, barberry has received much attention in *Pst* studies due to the increased interest in understating the genetics of the *Pst* pathogen. 

The *Pst* genome is highly heterozygous and contains 25,288 protein-coding genes. A 110-Mb draft sequence of a *Pst* isolate *CY32* was reported by Zheng et al. [28], using ‘fosmid-to-fosmid’ strategy. Re-sequencing analyses showed a high genetic diversity of six *Pst* isolates collected from different continents. The draft genome assembly, in association with transcriptomics, provides the first insight into the molecular biology of *Pst*. A large number of *Pst* populations of a diverse geographical origin were analyzed using microsatellite markers or simple sequence repeat (SSR) markers. The results showed a higher genotypic diversity, recombinant population structure and high sexual reproduction ability in the Himalayan and the neighboring regions (Nepal, Pakistan and China), the center of origin of *Pst* [11]. The virulence phenotyping and molecular genotyping approaches were used to study the genetic diversity of *Pst* populations in three epidemiological regions in China, and the results revealed that the Xinjiang region had a higher genetic diversity of *Pst* compared to the other epidemic regions [90]. However, these studies lack detailed sequence information of the fundamental genetic changes in pathogen populations. Whole-genomic and transcriptomic data into field pathogen surveys can help to understand this fundamental question [52]. It is easy to identify causative alleles of DNA sequence variations and changes in pathogen fitness using potential genome-guided techniques [35]. 

To date, the application of various molecular markers, such as amplified fragment length polymorphism (AFLP), fragment length polymorphism (FLP), random amplified polymorphic DNA (RAPD) and simple sequence repeats (SSR) have been used to understand the genetic diversity of *Pst* [87,88,89]. The whole-genome re-sequencing of several isolates offers an ideal resource for the development of SSR markers, which are useful for genetic and population studies of *Pst* [6]. The single nucleotide polymorphism (SNP) has been used as the standard genetic marker to identify disease-associated alleles. Using SNP genotyping technology, we can efficiently investigate genotype variation across 100,000–1,000,000 SNPs. Quantitative trait locus (QTL mapping) is an advantageous approach to construct linkage maps between the wheat stripe rust-resistance-genes and genetic markers. Molecular techniques have greatly increased the available markers in rust fungi for constructing linkage maps [18,67]. 

Due to the evolution of new virulent races, resistant cultivars may become susceptible after a few years in a particular region or country [40]. Every year new races of *Pst* are identified in wheat-growing areas in the world, especially in the US, China, India and Pakistan, among others [40,71,91,92]. The mechanism involved in the virulence diversity and genetic variation is essential to understand the role of the alternate hosts of the wheat stripe rust pathogen. Few studies have been reported on the genetics of *Pst* virulence. The first study of the genetics of *Pst* demonstrating virulence and avirulence genes and gene-for-gene relationship in the *Pst*-pathosystem was conducted by Wang et al. [93]. They selfed a US isolate of race *Pst*-127 on *B. vulgaris* and obtained 29 progeny isolates. They tested the isolates on *Yr* single-gene lines and found the parental isolate as homokaryotic (homozygous) for virulent loci to *Yr1*, *Yr2* and *Yr9* and for avirulence loci to *Yr5*, *Yr15*, *Yr24*, *Yr32* and *YrSP*. In contrast, segregation was observed for the virulence phenotypes (VPs) to *Yr6*, *Yr7*, *Yr8*, *Yr10*, *Yr17*, *Yr19*, *Yr27*, *Yr43*, *Yr44*, *YrExp1*, *YrExp2*, *YrTr1*, and *Yr76* (*YrTye*) in different ratios. The avirulence to seven *Yr* genes (*Yr6*, *Yr7*, *Yr8*, *Yr19*, *YrExp2*, and *YrTye*) was dominant and controlled by single genes in the parental isolate. In contrast, avirulence to *Yr17* and *YrExp1* in the parental isolate was controlled by individual recessive genes. Avirulence to *Yr44* was controlled by two independent dominant genes and avirulence to *Yr43,* and an unknown *Yr* gene was controlled by two recessive genes (7:9 ratios). Tian et al. [87] selfed a Chinese *Pst* isolate (*Pinglan 17-7*) on *B*. *shensiana* and obtained 118 progeny isolates. They found 24 virulence phenotypes (VPs), 82 multi-locus genotypes (MLGs) using 13 polymorphic SSR markers. A preliminary linkage map was constructed with eight of 24 avirulence/virulence loci and 10 SSR markers. Avirulence to *Yr4*, *Yr32* and *Yr44* in the parental isolate was controlled by two recessive complementary genes (1:15 ratios). They found that a highly diverse population of *Pst* could be generated by selfing a single isolate on barberry and this progeny population could be used for its virulence characterization. In another study using the same method, 120 progeny isolates were obtained by selfing another Chinese isolate. They tested them on 25 *Yr* single-gene lines and found 51 VPs and 55 MLGs using 11 polymorphic SSR markers. Another linkage map was constructed using four avirulence loci and 11 SSR markers [88]. Rodriguez-Algaba et al. [56] studied the genetic diversity within and among aecia of *Pst* produced on *B*. *vulgaris*. The genetic markers confirmed the segregation and it resulted that the progeny isolates were derived from the parental isolate through sexual reproduction. 

Recently Mehmood et al. [94] generated 115 progeny isolates, using a Pakistani dominant race (*574232*) of *Pst*, through sexual reproduction on susceptible Himalayan *B*. *pseudumbellata*. The isolates were characterized using 24 wheat *Yr* single-gene lines and ten simple sequence repeat (SSR) markers. From the 115 progeny isolates, 25 virulence phenotypes (VPs) and 60 multilocus genotypes were identified. The parental and all progeny isolates were avirulent to *Yr5*, *Yr10*, *Yr15*, *Yr24*, *Yr32*, *Yr43*, *YrSp*, *YrTr1*, *YrExp2*, *Yr26*, and *YrTye* and virulent to *Yr1*, *Yr2*, *Yr6*, *Yr7*, *Yr8*, *Yr9*, *Yr17*, *Yr25*, *Yr27*, *Yr28*, *YrA*, *Yr44*, and *Yr3*. Based on the avirulence/virulence phenotypes, they found that VPs to *Yr1*, *Yr2*, *Yr9*, *Yr17*, *Yr47*, and *YrA* were controlled by one dominant gene; those to *YrSp*, *YrTr1*, and *Yr10* by two dominant genes; and those to *YrExp2* by two complementary dominant genes. Similarly, Wang et al. [95] studied the genetics of the *Pst* population obtained by selfing a Chinese predominant race *CYR32* on *B*. *aggregate*. They found 27 VPs and 65 MLGs. A linkage map of 10 virulence/avirulence genes was constructed using 10 SSR markers. The results indicated a complex interaction between the virulence genes in the pathogen and the avirulence genes in the host wheat lines. Yuan et al. [89] generated a segregating population of 119 isolates by the self-fertilization of *Pst* isolate *08-220* (race PSTv-11) on barberry leaves under controlled greenhouse conditions. They constructed a genetic map of six linkage groups using a massive amount of genotype-by-sequencing (GBS) SNP markers. All these studies reported the dominance and recessiveness of the avirulence or virulence genes. These results may help to understand the genetic diversity of *Pst*, the role of sexual reproduction, host–pathogen interaction, and selection of resistance genes in breeding programs to some extent. However, large scale surveys in the areas of high genetic diversity, like the Himalayan region in Pakistan and several Provinces in China, are needed to study the factors involved in the genetic diversity of the *Pst* populations. Several novel technologies such as auto-fluorescent proteins in combination with confocal laser scanning microscopy (CLSM), single-molecule detection, atomic force microscopy, and differential fluorescence induction (DFI) combined with optical trapping (OT) [96] can be used to study the microbial behavior of *Pst*.

During the infection process, the pathogens deliver virulence-associated “effector” proteins to promote plant susceptibility. However, little is known about the effector proteins and their functions in the *Pst*–wheat association. Zhao et al. [97] reported a candidate effector *Pst*_8713 isolated based on the genome data of CY32 and the expression of *Pst_8713* was highly induced during the early infection stage. Cheng et al. [98] reported a highly induced candidate effector from *Pst*, PSTha5a23, which shows a low level of intra-species polymorphism and it plays an important role in plant defense suppression and rust pathogenicity. They also highlighted the utility of gene overexpression in plants as a tool for studying effectors from *Pst*. Liu et al. [99] identified a *Pst* effector candidate (PEC6) with a pattern-triggered immunity (PTI) suppression function and its corresponding host targets in a host species-independent manner and interacts with adenosine kinases. Yin et al. (2014) [100] reported enhanced level of auxins during the infection of wheat by the stem rust pathogen (*Pgt*) and they observed a correlation between the enhanced auxin levels and the *Pgt* gene (*Pgt*-IaaM) that encodes a putative tryptophan 2-monooxygenase protein in haustoria. Cantu et al. [27] identified five *Pst* candidate effectors from haustorial expressed secreted proteins with polymorphism by resequencing genomes from four US and UK *Pst* isolates. Because *Pst* lacks a stable and efficient transformation system, few *Pst* effectors have been studied at the function level [101]. Garnica et al. [102] purified *Pst* haustoria and used next-generation sequencing platforms to assemble the haustorial transcriptome as well as the transcriptome of germinated spores and confirmed the expression patterns of 94 potential *Pst* effector candidates through RT-PCR analysis. Yin and Hulbert [103] reported the use of bacterial type three secretion systems (TTSS) to deliver proteins into wheat cells, which is feasible for studying the functions of *Pst* effectors. Similarly, an extensive polymorphism in *Pgt* candidate effector genes was observed during a comparative study of the genomics of Australian isolates of the wheat stem rust pathogen [104]. More research is expected on *Pst* effectors, to better understand the function of these effectors in *Pst*–wheat interaction.

## 9. Conclusions

Despite the advancements in genetic techniques and all the previous research work discussed in this review, there are still a lot of questions for plant geneticists to answer in further studies. Several *Berberis* and *Mahonia* species have been known as susceptible hosts to *Pst* and more susceptible hosts are likely to be identified in the future. Alternate hosts of *Pst* help the pathogen survive adversarial environmental conditions and provide new inoculum through sexual reproduction to infect wheat, other cereals and grasses. They are essential facilitators to *Pst,* and they provide shelter for the survival of the pathogen; provide initial inoculum for the wheat rust disease epidemics under favorable conditions; diversify pathogen populations through sexual reproduction, and may cause the emergence of more virulent races of *Pst*. The phenology of the alternate hosts and ecological conditions also play an essential role during the disease infection process by *Pst*. It is needed to study the molecular mechanism involved in pathogenicity, *Pst* effector proteins, and the susceptibility of the alternate hosts under natural conditions. In the future, we need to know about the genetic frequencies and the identification of effectors within *Pst* populations to control the wheat stripe rust pathogen durably [25]. Regardless of the importance of alternate hosts, the most important thing is to cultivate resistant cultivars to control *Pst* through the identification of both resistant genes of wheat cultivars and the virulent races of the pathogen. To avoid the spread of the wheat stripe rust disease epidemics and their economic impacts in wheat-growing countries, intensive monitoring with the fast and reliable identification of virulence phenotypes and resistant genotypes is essential. For this, it is much needed to invest in the development of resistant cultivars to combat the pathogen rather than devising a less-needed chemical/fungicide to slow the spread of the virulent races of *Pst*. Moreover, a strong collaboration is needed, as soon as reasonably practical, among the research scientists of different wheat-growing regions, countries and continents to fight against this enemy of humanity. 

## Figures and Tables

**Table 1:** *Berberis* and *Mahonia* spp. susceptible to the wheat stripe rust pathogen, *Puccinia striiformis* f. sp. *tritici*.

Sr. No.	Species	No.	Infection type	Country	References
1	*B.chinensis*, *B. holstii, B. koreana, B. vulgaris, B. thunbergii*	5	Artificial	US	[13]
2	*Mahonia aquifolium*	1	Artificial	China	[22]
3	*B. atrocarpa*, *B. stenostachya*, *B. soulieana*, *B. shensiana*, *B. wangii*, *B. phanera*, *B*. *davidii*, *B. poiretii*, *B. aggregata* var*. integrifolia*, *B. potaninii*, *B*. *jamesiana*, *B. ferdinandi-coburgii*, *B. brachypoda*, *B. circumserrata*, *B. platyphylla*, *B. dasystachya*, *B. aggregata*, *B. guizhouensis*	18	Artificial	China	[7,15]
	*B. brachypoda*, *B. soulieana*, *B. shensiana*, *B. potaninii*	4	Artificial + Natural		
4	*B. franchetiana var. glabripes*, *B. gyalaica*, *B. jaeschkeana* var. *bimbilaica*, *B. kongboensis*, *B. wilsonae*, *B. zanlanscianensis*	6	Artificial	China	[18]
5	*B. lycium, B. orthobotrys*, *B*. *pseudumbellata*, *B. stewartiana*, *B. brandisiana*, *B. pseudumbellata* subsp. *pseudumbellata*, *B. pseudumbellata* subsp. *gilgitica*	7	Artificial	Pakistan	[12]
6	*B. heteropoda*, *B. nummularia*, *B. kaschgarica*	3	Artificial	China	[61]
7	*B. approximate*, *B. dictyoneura*, *B. dubia*, *B. kansuensis*, *B. vernae*, *B. aggregate*, *B. circumserrata*, *B. dasystachya*, *B. diaphana*, *B. poiretii*	10	Artificial	China	[62]
